# Supplementary material for: Angiotensin-II-Evoked Ca2+ Entry in Murine Cardiac Fibroblasts Does Not Depend on TRPC Channels
Source: Cells. 2020 Jan 29;9(2):322. doi: 10.3390/cells9020322 (PMC7072683; doi:10.3390/cells9020322)
Supplement: Supplementary file 1 [file cells-09-00322-s001.pdf]

SUPPLEMENTARY MATERIAL

Angiotensin-II-evoked Ca^{2+} entry in murine cardiac fibroblasts does not depend on TRPC channels

Juan E. Camacho Londoño[1,2,†*], André Marx[1,†], Axel E. Kraft[1,2], Alexander Schürger[1,2], Christin Richter[1], Alexander Dietrich[2], Peter Lipp[4], Lutz Birnbaumer[5] and Marc Freichel[1,2,*]

[1]Pharmakologisches Institut, Ruprecht-Karls-Universität Heidelberg, INF 366, 69120 Heidelberg, Germany.
[2]DZHK (German Centre for Cardiovascular Research), partner site Heidelberg/Mannheim, Germany.
[3]Walther-Straub-Institut für Pharmakologie und Toxikologie, Ludwig-Maximilians-Universität, 80336 München, Germany.
[4]Medical Faculty, Centre for Molecular Signalling (PZMS), Institute for Molecular Cell Biology and Research Center for Molecular Imaging and Screening, Saarland University, Homburg/Saar, Germany.
[5]Laboratory of Neurobiology, NIEHS, North Carolina, USA and Institute of Biomedical Research (BIOMED), Catholic University of Argentina, Buenos Aires, Argentina.
*Correspondence: juan.londono@pharma.uni-heidelberg.de and marc.freichel@pharma.uni-heidelberg.de
[†]These authors contributed equally to this work.

Graphical Abstract: Angiotensin-II-evoked Ca^{2+} entry in murine cardiac fibroblasts (CFs) does not depend on TRPC channels.

Figure S1. AngII- and Thrombin- induced Ca^{2+} transients in TRPC1/C4-DKO cardiac fibroblasts.

Figure S2. AngII-induced Ca^{2+} release and Ca^{2+} entry in the absence of TRPC3/C6 or after TGF-β pre-treatment.

Figure S3. AngII-induced Ca^{2+} release and Ca^{2+} entry in cardiac fibroblasts after acute blockage with the TRPC blocker T320722.

Figure S4. AngII-induced Ca^{2+} release and Ca^{2+} entry in cardiac fibroblasts in the absence of all seven TRPC proteins.

Table S1. Primers used for qPCR analysis of *Trpc* transcripts.

Table S2. Immunocytochemistry conditions used for characterization and analysis of cardiac fibroblasts.

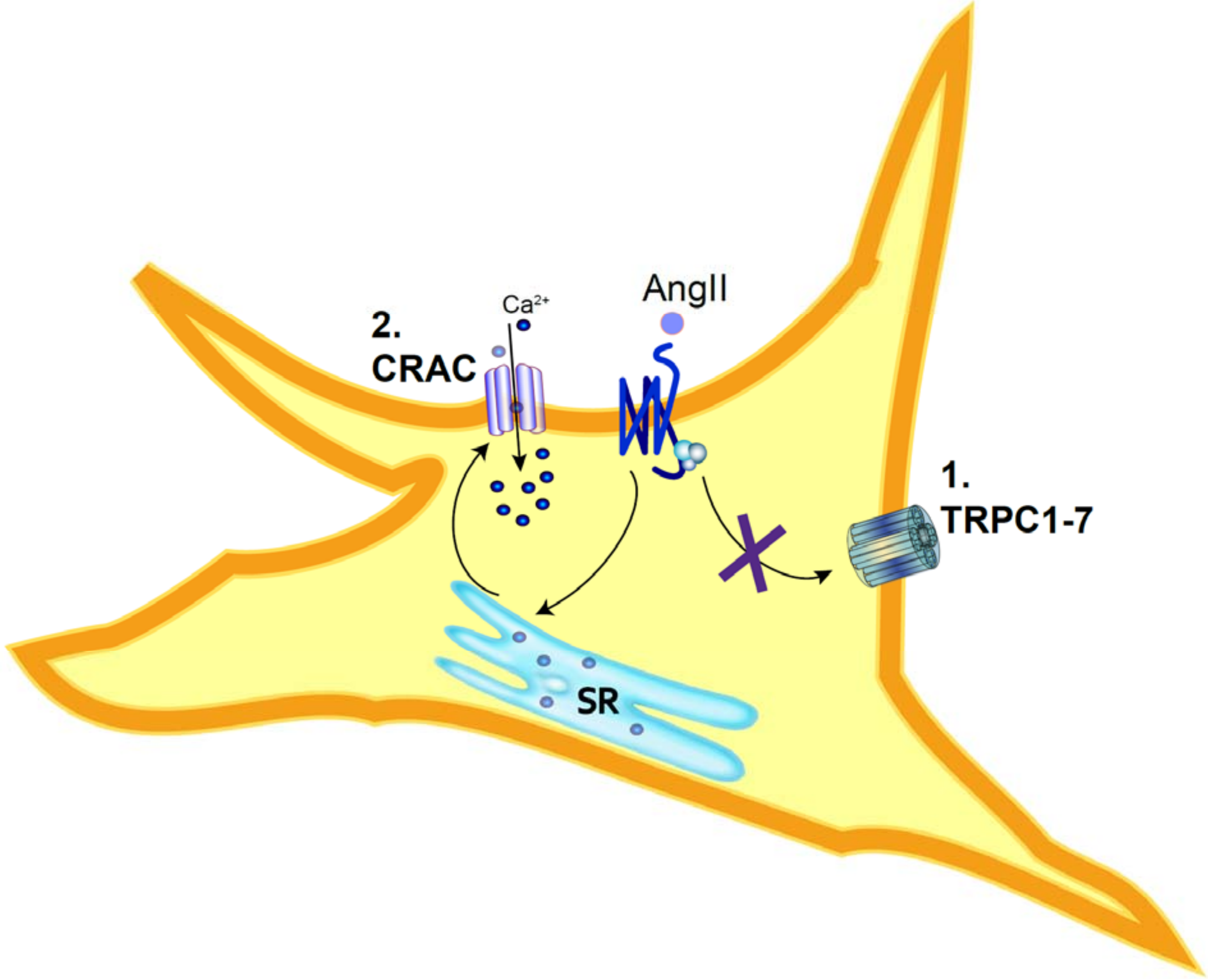

Graphical Abstract: Angiotensin-II-evoked Ca^{2+} entry in murine cardiac fibroblasts (CFs) does not depend on TRPC channels. Using genetic and pharmacological tools we evaluated the Angiotensin II (AngII)-induced Ca^{2+} release and Ca^{2+} entry. We concluded: 1. that complete deletion of all 7 TRPC proteins does not alter this acute response to AngII, and 2. that GSK7975A, a CRAC blocker, was able to abolish the AngII-induced Ca^{2+} entry in CFs.

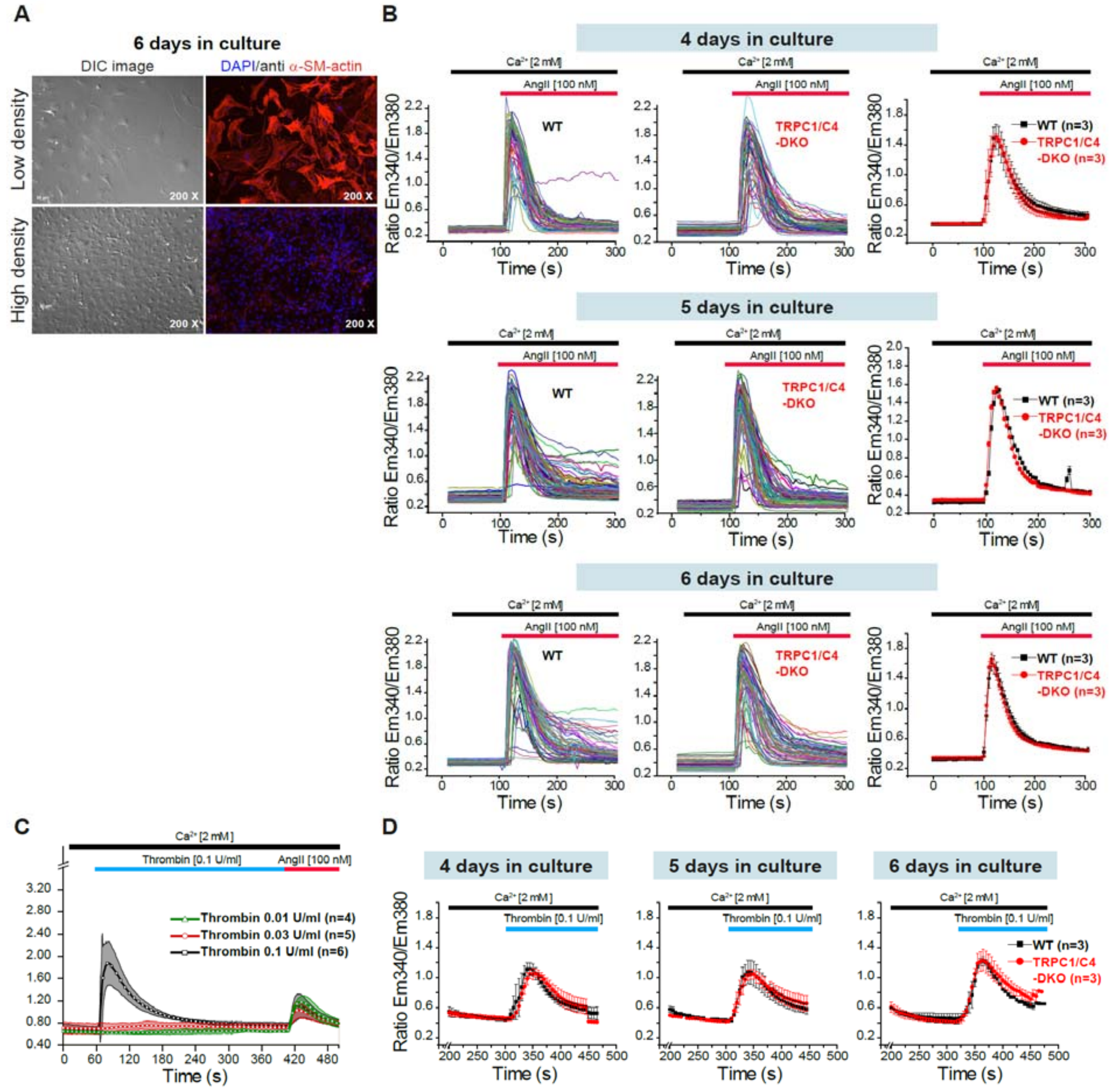

Figure S1. AngII- and Thrombin- induced Ca^{2+} transients in TRPC1/C4-DKO cardiac fibroblasts. **(A)** Analysis of α-smooth muscle actin after 6 days in culture from WT cardiac fibroblasts cultivated at low and high density conditions. **(B)** AngII-induced Ca^{2+} transients in primary cardiac fibroblasts from WT (black) and TRPC1/C4-DKO (red) mice. Ca^{2+} transients were measured in the presence of 2 mM extracellular Ca^{2+}. Left panels: Original traces and right panels: Mean values of three independent preparations (hearts). **(C)** Ca^{2+} transients in WT fibroblasts induced by different concentrations of Thrombin. **(D)** Thrombin-induced Ca^{2+} transients in primary cardiac fibroblasts from WT (black) and TRPC1/C4-DKO (red) mice. Ca^{2+} transients were measured in the in the presence of 2 mM extracellular Ca^{2+}. n= number of independent preparations (hearts). All cells were cultured at high density.

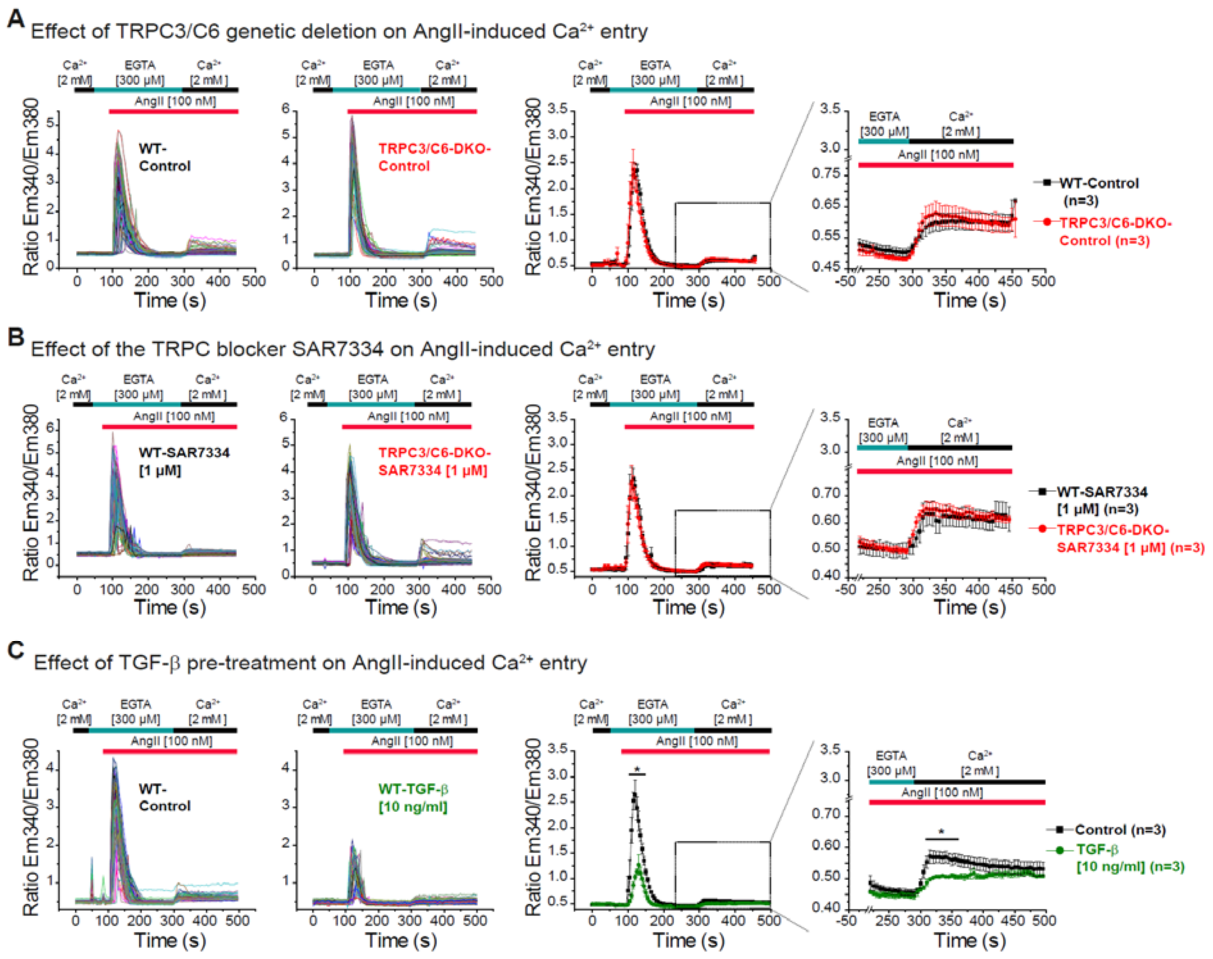

Figure S2. AngII-induced Ca^{2+} release and Ca^{2+} entry in the absence of TRPC3/C6 or after TGF-β pre-treatment. (A) AngII-induced Ca^{2+} release and Ca^{2+} entry in primary CFs from WT (black) and TRPC3/C6-DKO (red) mice. Ca^{2+} release was measured in the absence of extracellular Ca^{2+} (300 µM EGTA) and Ca^{2+} entry was monitored in the presence of 2 mM extracellular Ca^{2+}. Left panels: Original traces and right panels: Mean values from three independent preparations (hearts). **(B)** Measurements performed as in (**A**) but in cells pre-incubated (10 min) with the TRPC3/C6/C7 antagonist SAR7334 (1 µM). **(C)** AngII-induced Ca^{2+} release and Ca^{2+} entry in primary CFs from WT mice cultivated in the presence of 10 ng/ml TGF-β (green) or under control conditions (black). Left panels: Original traces from Ca^{2+} measurements and right panels: Mean values from 3 independent preparations. n= number of independent preparations (hearts). All cells were analyzed 6 days after isolation and were cultured at high density. *$p< 0.05$ according to the unpaired Student's t-test.

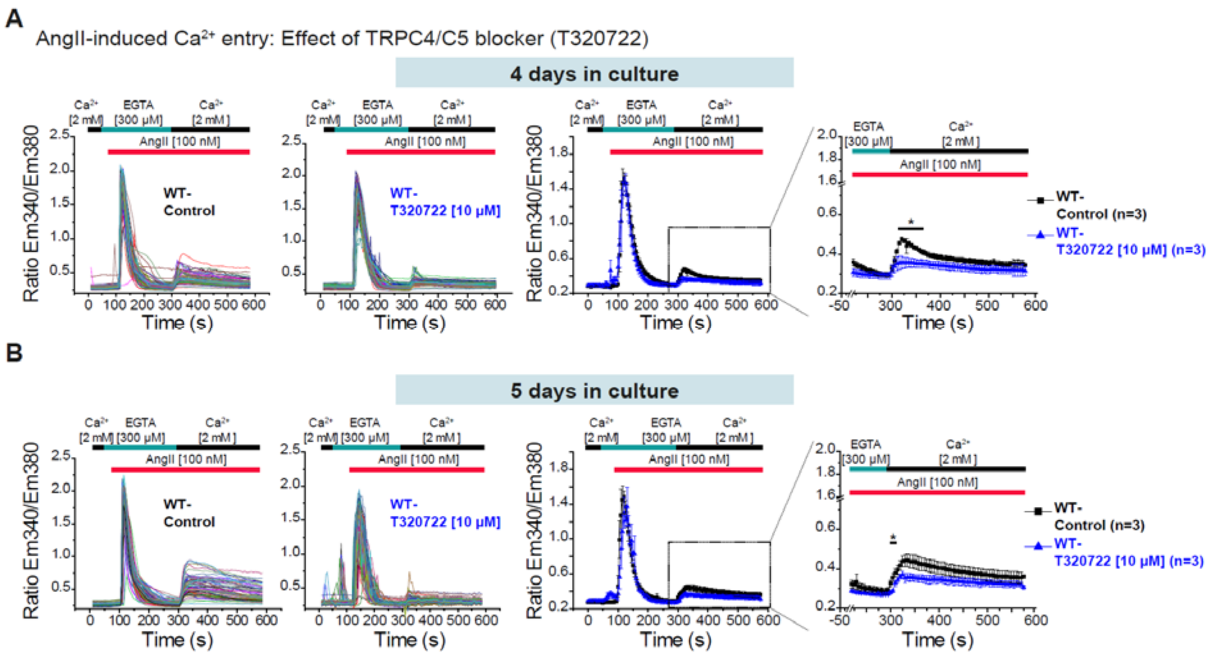

Figure S3. AngII-induced Ca^{2+} release and Ca^{2+} entry in cardiac fibroblasts after acute blockage with the TRPC blocker T320722. (A) AngII-induced Ca^{2+} release and Ca^{2+} entry in primary cardiac fibroblasts from WT mice pre-incubated (10 min) with 10 µM of the TRPC4/C5 blocker T320722. Ca^{2+} release was measured in the absence of extracellular Ca^{2+} (300 µM EGTA) and Ca^{2+} entry was monitored in the presence of 2 mM extracellular Ca^{2+}. Left panels: Original traces and right panels: Mean values of three independent preparations (hearts); cells were analyzed after 4 **(A)** or 5 days **(B)** of isolation and were cultured at high density. n= number of independent preparations (hearts). *$p < 0.05$ according to the unpaired Student's t-test.

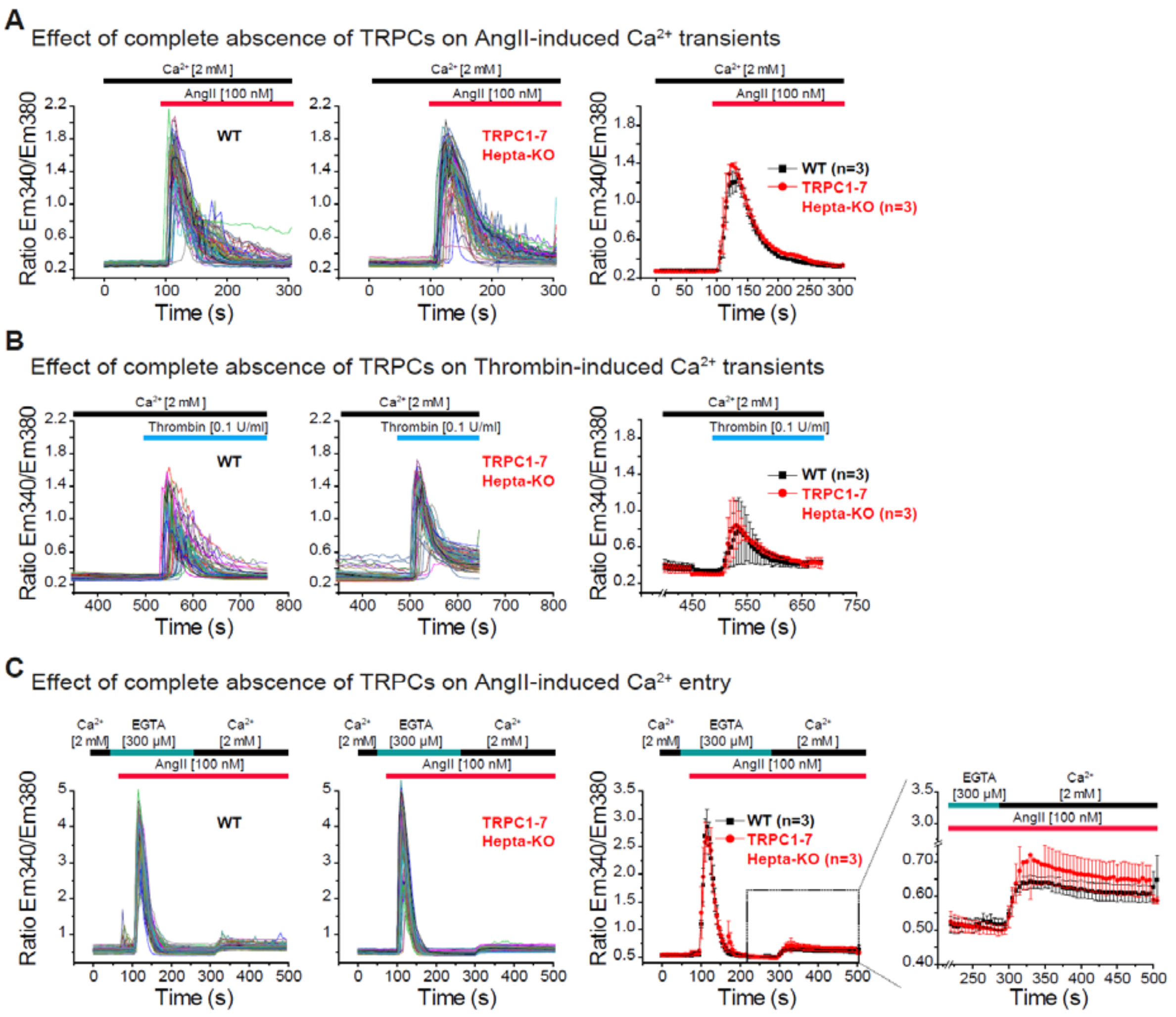

Figure S4. AngII-induced Ca^{2+} release and Ca^{2+} entry in cardiac fibroblasts in the absence of all seven TRPC proteins. (A) AngII- and **(B)** thrombin-induced Ca^{2+} transients in primary CFs from WT (black) and TRPC-hepta (*Trpc1/2/3/4/5/6/7*$^{-/-}$) KO (red) mice. Ca^{2+} transients were measured in the presence of 2 mM extracellular Ca^{2+}. Left panels: Original traces and right panels: Mean values of three independent preparations (hearts). **(C)** AngII-induced Ca^{2+} release and Ca^{2+} entry in primary CFs from WT (black) and TRPC-hepta KO (red) mice. Ca^{2+} release was measured in the absence of extracellular Ca^{2+} (300 µM EGTA) and Ca^{2+} entry was monitored in the in the presence of 2 mM extracellular Ca^{2+}. Left panels: Original traces and right panels: Mean values of three independent preparations. n= number of independent preparations (hearts). All cells were analyzed 6 days after isolation and were cultured at high density.

Table S1. Primers used for qPCR analysis of *Trpc* transcripts. Primers sequences and probe number for from the Universal Probe Library (Roche).

Primer name	Primer sequence	Probe number
TRPC1 fw	ctgaaggatgtgcgagaggt	63
TRPC1 rev	cacgccagcaagaaaagc	
TRPC2sv1 fw	gtgtggatcgagggcttg	31
TRPC2sv1 fw	acaggatgaccacgtccag	
TRPC2sv2 fw	tccttgtcttcctcggagtc	52
TRPC2sv2 fw	ttcacagatagggcactggac	
TRPC3 fw	ggtgaactgaaagaaatcaagca	19
TRPC3 rev	cgtcgcttggctcttatctt	
TRPC4 fw	aaacttttggttcagaaaggtgtc	104
TRPC4 rev	acagttacagcggacctcgt	
TRPC5 fw	ggcataaaagtcatcttgctgaa	10
TRPC5rev	gctaagcagaagttccacagc	
TRPC6 fw	aggcaaaaggttagcgacaa	20
TRPC6 rev	ggcataaaagtcatcttgctgaa	
TRPC7 fw	aatggcgatgtgaacttgc	77
TRPC7 rev	gtttgattcggctcagacttg	
H3F3A fw	gccatctttcaattgtgttcg	19
H3F3A rev	agccatggtaaggacacctc	
AIP fw	accagtcatccaccaagagg	66
AIP rev	aggcgatggcgtcatagta	
CXCC1 fw	tagtgccgaccgctgact	26
CXCC1 rev	ggcctctcccctaactgaat	

Fw: Forward, rev: reverse.

Table S2. Immunocytochemistry conditions used for characterization and analysis of cardiac fibroblasts.

	anti-P4HB	anti-DDR2	anti-α-actinin	anti-α-SMA	anti-CD31
**positive control**	fibroblasts	fibroblasts	cardiomyocytes	iSMC	MAEC
**acetone permeabilization**	yes	yes	yes	yes	no
**blocking**	1 % BSA in PBST	1 % BSA in PBST	1 % BSA in PBST	1 % BSA in PBST	1 % BSA in PBS
**concentration**	1 µg/ml (in PBST)	1 µg/ml (in PBST)	150 µg/ml (in PBST)	10 µg/ml (in PBST)	10 µg/ml (in PBS)
**incubation time**	1 h	1 h	2 h	2 h	2 h
**secondary antibody**	anti-rabbit AlexaF488	anti-goat FITC	anti-mouse AlexaF594	anti-rabbit AlexaF488	anti-mouse AlexaF594
**2nd-ab provider**	Invitrogen (A11008)	Sigma-Aldrich (F9012)	Invitrogen (A11005)	Invitrogen (A11008)	Invitrogen (A11005)
**2nd-ab dilution**	1:1000 (in PBST)	1:200 (in PBST)	1:200 (in PBST)	1:200 (in PBST)	1:200 (in PBS)
**2nd-ab incubation time**	1 h	1 h	1 h	1 h	1 h

SMA: Smooth muscle actin, iSMC: ileum smooth muscle cells, MAEC: Mouse aortic endothelial cells, PBS: Phosphate buffered saline, PBST: PBS-Tween 20 and Alexa-F: Alexa Fluor.
